# Change of Acoustic Emission Characteristics during Temperature Induced Transition from Twinning to Dislocation Slip under Compression in Polycrystalline Sn

**DOI:** 10.3390/ma15010224

**Published:** 2021-12-28

**Authors:** Lajos Daróczi, Tarek Yousif Elrasasi, Talaye Arjmandabasi, László Zoltán Tóth, Bence Veres, Dezső László Beke

**Affiliations:** 1 Department of Solid State Physics, University of Debrecen, P.O. Box 400, H-4002 Debrecen, Hungary; lajos.daroczi@science.unideb.hu (L.D.); tarekyousif75@gmail.com (T.Y.E.); talaye.arjmand@gmail.com (T.A.); toth.laszlo@science.unideb.hu (L.Z.T.); tweener994@gmail.com (B.V.); 2 Department of Physics, Faculty of Science, Benha University, Benha 13518, Egypt

**Keywords:** stress/strain measurements, acoustic emission, Sn, plasticity, twinning, dislocation slip

## Abstract

In this study, acoustic emission (AE) measurements on polycrystalline tin as a function of temperature at different driving rates under compression were carried out. It is shown that there is a definite difference between the acoustic emission characteristics belonging to twinning (low temperatures) as well as to dislocation slip (high temperatures). The stress averaged values of the exponents of the energy probability density functions decreased from ε = 1.45 ± 0.05 (−60 °C) to ε = 1.20 ± 0.15 (50 °C) at a driving rate of $\varepsilon =0.15\;s^{-1}$, and the total acoustic energy decreased by three orders of magnitude with increasing temperature. In addition, the exponent *γ* in the scaling relation *S_AE_~D_AE_^γ^* (*S_AE_* is the area and *D_AE_* is the duration) also shows similar temperature dependence (changing from *γ* = 1.78 ± 0.08 to *γ* = 1.35 ± 0.05), illustrating that the avalanche statistics belong to two different microscopic deformation mechanisms. The power law scaling relations were also analyzed, taking into account that the detected signal is always the convolution of the source signal and the transfer function of the system. It was obtained that approximate values of the power exponents can be obtained from the parts of the above functions, belonging to large values of parameters. At short duration times, the attenuation effect of the AE detection system dominates the time dependence, from which the characteristic attenuation time, *τ_a_*, was determined as *τ_a_* ≅ 70 μs.

## 1. Introduction

It is well known that processes taking place during plastic deformations have intermittent character and avalanches of acoustic emission (AE) signals are often emitted during these processes [1,2,3,4,5,6,7,8,9,10]. It is also known that during plastic deformation the dominant sources of AE are both the collective motions of dislocations as well as deformation twinning [1,2,3,4,5,6,7,8,9], and that the dislocation glide avalanches and intermittent twinning events can have different AE characteristics (see e.g., [1,6]). Furthermore, the transition between the above two deformation mechanisms is often observed, especially in hcp metals [1,2,3,7,11], and this transition depends on many factors, such as the temperature [11,12,13,14], grain size [8,15], type of the load (compressive or tensile) [3,13], the deformation rate [11], and crystallographic orientation [3].

In tin, the dominant deformation mechanism at low temperatures and low deformation rates is deformation twinning. With increasing temperature it gradually changes to the mechanism of collective motion of dislocations (see e.g., [2]). Although, it was already known earlier that AE indeed reflects the intermittent character of plastic deformations, real statistical analysis of AE signals, collected during plastic deformation, started only at the beginning of this century [3]. Thus, it was shown by numerical simulations and experiments on creeping ice [3] that dislocations organize into scale free patterns of avalanches, characterized by damped power law distributions [16,17]:

(1) $$P\left(x\right)\;\approx \;x^{-\xi }e^{-\frac{x}{x_{c}}}$$

where *P*(*x*) is the probability density of a given quantity, *x*, the energy, size, or duration time of individual AE noise peaks, *ξ* the characteristic exponent, and *x_c_* the cutoff value.

It was found that the energy exponent, *ε*, for dislocation avalanches was *ε* = 1.5 ± 0.1 in ice single crystal [18] and in Cd and Zn (0.08% Al) single crystals [2]. On the other hand, although it was found in [2] that the AE waveforms of slip and twinning events were different, the energy distributions constructed from AE signals of the above two typical deformation modes were the same (i.e., the energy exponents were the same) and no distinction was possible on this basis. This led the authors to conclude that both phenomena belong to the same global dynamics. Later on, the same group published a different value of *ε* (*ε* = 1.6 ± 0.1) [19], but again argued that the same exponent supports the scale-free, intermittent character of plasticity (see also [20]). In more recent papers [21,22] the above picture was refined by illustrating that the exponents did not take on a universal value (e.g., there is a definite difference of the energy exponents in hcp and fcc structures [22]) and that the exponents showed a sensitivity to the microstructure (grain size) too [21]. Thus, it was concluded in [22] that *ε* = 1.40 ± 0.03 for ice, *ε* = 1.45 ± 0.05 for Cd, *ε* = 2.00 ± 0.05 for Al as well as *ε* = 1.54 ± 0.08 for CuAl alloys. In addition, it was also shown in [22] that there is a crossover between the intermittent and continuous regimes of plastic flow. Nevertheless, although there are illustrations in the literature that a distinction between the dislocation glide avalanches and twinning is possible on the basis of the AE waveforms [2], the question about the resolution of the possible difference (e.g., between the characteristic exponents for the above two deformation mechanisms in the same material) is still open. In addition, in recent papers [10,23] it was also pointed out that, although the existence of ubiquitous, universal scaling exponents would suggest the use of simple analytical models (such as the mean field models [24]), the plastic flow can have different character depending on the microstructural specifics.

In this paper, we report AE measurements on polycrystalline tin as a function of the temperature at different driving rates under compression. First, we demonstrate that there is a definite difference between the acoustic emission characteristics belonging to twinning as well as to dislocation slip at $\varepsilon =0.15\;s^{-1}$ driving rate. For example, the energy exponents decrease from 1.45 ± 0.05 (at −60 °C) to 1.20 ± 0.15 (at 50 °C). In addition, we show that the total acoustic energy, as expected, also has a transition from large to small values (changing by about three orders of magnitude) with increasing temperature. The temperature dependence of the AE activity curves reflects similar crossover between the activity belonging the low temperature twinning and high temperature slips.

The theoretical results [16,17,23,24] predict that, for example, the slope of the avalanche size ($S=\int_{o}^{D}\left|U\left(t\right)\right|dt$ (where *D* is the duration time of the avalanche and $\left|U\left(t\right)\right|$ is the absolute value of the voltage signals detected, this being proportional to the velocity of the elemental plastic shift and related to the motion of dislocations or deformation twinning, *V(t)*) versus *D (S~D^γ^*))), *γ*, should be different for different universality classes (i.e., for example for different deformation mechanisms). The characteristic exponent *τ*, belonging to the area distribution (*P(S)~S^−τ^*) has a similar behavior; even in mean filed approximation, *τ* ≅ *γ* is predicted [24]. This also means that the temporal shapes of avalanches [10,23,24,25,26,27,28,29] (i.e., the *V(t)* function), on a properly reduced scales, are universal within a class of materials with the same deformation mechanism. Indeed, we will show that, similarly to *ε, γ* also changes: *γ* = 1.78 ± 0.08 for twinning and *γ* = 1.35 ± 0.05 for dislocation slip mechanism, respectively, confirming that these characteristic exponents are different for the two deformation mechanisms. Since the transition between the two mechanisms can have a driving rate dependence [11,24,25], we also carried out additional investigations at four smaller driving rates (between 0.005 s^−1^ and 0.025 s^−^^1^). We also analyzed the power law-type scaling relations between the energy, amplitude, size and duration times of AE signals, also taking into account the distortion effect of the AE device. It will be demonstrated that, in accordance with [10,29,30,31], reliable approximate estimates of the exponents of the above scaling relations can be obtained only at large values of the parameters while, for example, at short duration times the attenuation effects of the AE detecting device dominate.

## 2. Experimental

The polycrystalline Sn samples were produced by the following method: Sn flakes of analytical purity were melted and snuffed up into a 3 mm internal diameter glass tube. After solidification, the glass mold was carefully cracked; samples have been cut from the cast tin rods by spark erosion. The grain size was 50 μm. The stability of the white β-Sn at low temperatures was good; we did not observe the formation of grey α-Sn during the relatively short measurements.

**Figure 1:**
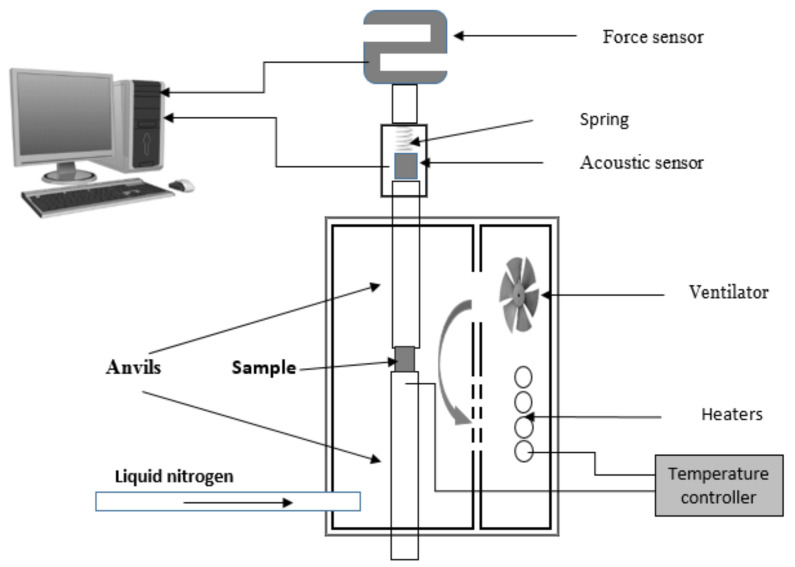
Experimental arrangement.

Acoustic emission measurements were performed during compression tests between −60 °C and +90 °C. A schematic of experimental arrangement is shown in Figure 1. The acoustic sensor was connected to the higher anvil. The sample was heated by a resistance tube shaped heating element. The cooling was controlled by pumping liquid nitrogen into the cavity of the heating chamber. The temperature was measured by a copper-constantine thermocouple embedded into the lower anvil quite close to the sample. The whole arrangement was mounted on a Chatillon TCD 225 tensile test console (Ametek Inc., Berwyn, PA, USA) producing a constant deformation rate $\varepsilon =0.15\;s^{-1}$ under displacement control. The original dimensions of the cylindrical tin samples were 3 mm diameter and 3 mm height. After deformation, the height of samples was 1 mm. The acoustic signals were detected by a Micro-100S (Physical Acoustic Corporation, Princeton Junction, NJ, USA) piezoelectric sensor connected by a long steel waveguide to the sample to protect it from the widely variable temperatures (the temperature of the sample can be as low as −60 °C, or as high as 100 °C [32,33]. The setup has a homemade 60 dB grounded base amplifier, with very good transmissibility in the 0 Hz–200 kHz frequency range. The signals were recorded using a National Instruments PCI-6111 multifunction data acquisition board (National Instruments, Austin, TX, USA), with a 5 MS/s/channel sampling rate. In our evaluation program, a filtering was incorporated. Signals between 30 kHz and 1 MHz were further processed.

In the second set of experiments, in order to investigate the effect of the driving rate, measurement at four lower driving rates (between 0.005 s^−1^ and 0.025 s^−1^) were carried out with the same sampling rate (5 MS/s/channel) at different temperatures on the same sample in the same setup.

## 3. Results and Discussion

### 3.1. Results at $\dot{\varepsilon }$ = 0.15 s^−1^ Deformation Rate

**Figure 2:**
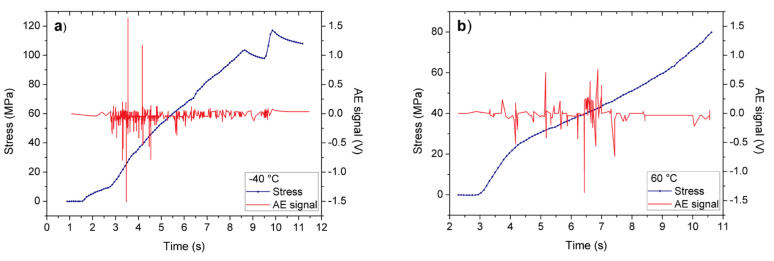
Stress-strain curve (blue curves: the strain is proportional to the time) and the acoustic emission events (voltage of the piezoelectric sensors), detected simultaneously in polycrystalline Sn samples (**a**) at −40 °C and (**b**) at 60 °C.

Figure 2a,b shows the applied stress and the emitted acoustic signals, detected simultaneously, as a function of time at −40 °C and 60 °C, respectively. One can see that there is a change in the slope of the plastic part, as well as in the number of acoustic noise hits. For the detection of the acoustic signals, the threshold value was 0.04 V and the hit definition time (HDT) was chosen to be 80 μs well as the AE amplitude was defined as the peak voltage.

**Figure 3:**
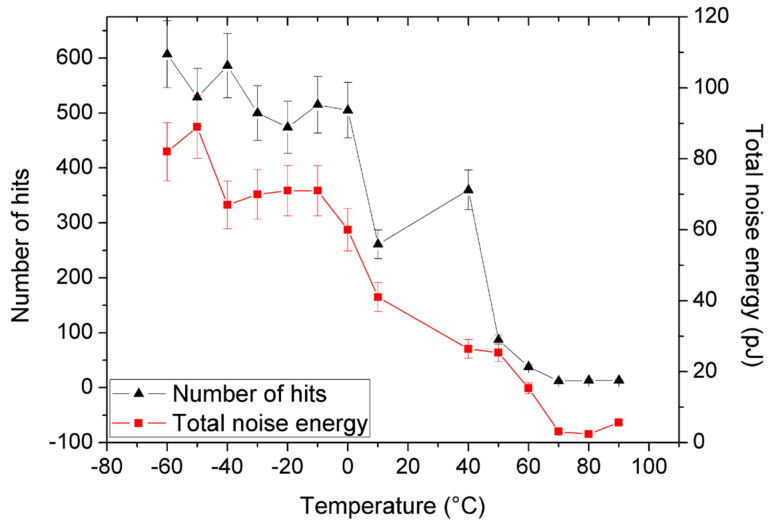
Temperature dependence of the total number of acoustic events and the total noise energy.

The total number of events and the total noise energy of the deformation process as a function of temperature can be seen in Figure 3. These quantities show again a similar characteristic temperature dependence. The energy of an individual acoustic event, *E_i_*, was determined from the integration of the voltage versus time signal (*U(t)*) [34,35,36,37]: $E_{i}≅\frac{1}{R}\int_{t_{i}}^{t_{i}+D_{i}}U^{2}\left(t\right)dt$, where *R* is a reference electrical resistance [35,36] (taken as 1 MΩ), and *D_i_* is the duration time of the event. The total noise energy is given by $E=\sum_{i}E_{i}$. It is worth adding that the AE energy shows a definite correlation with the plastic strain energy too [38].

**Figure 4:**
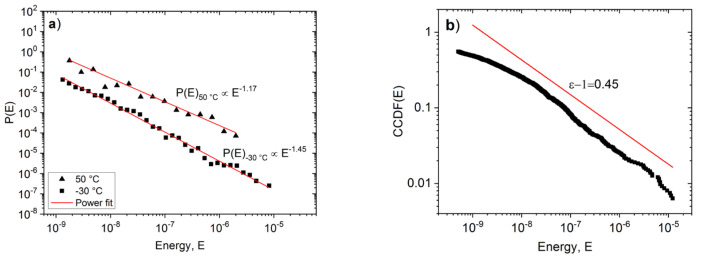

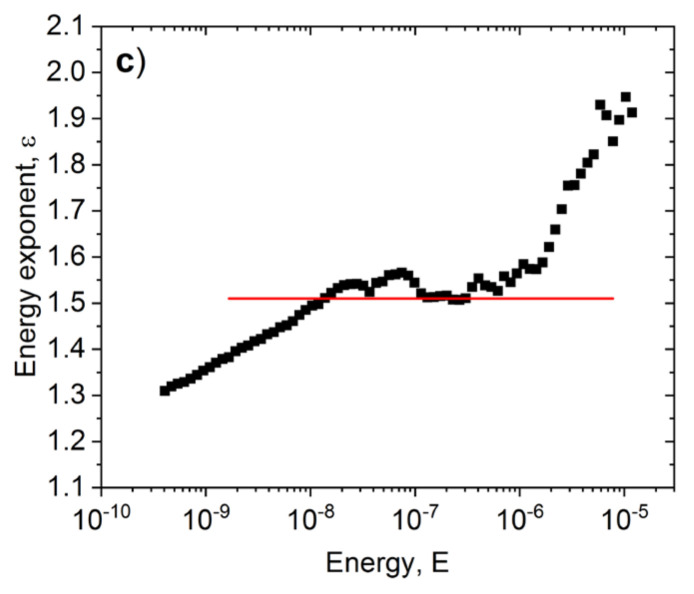
(**a**) Energy probability density functions at −30 °C and 50 °C, (**b**) the corresponding CCDF function at −30 °C, (**c**) maximum likelihood estimation at −30 °C.

Figure 4 show, as an illustration, the energy probability density functions, PDF, (*P*(*E*)~*E*^−*ε*^) at −30 °C and 50 °C, respectively. In our experiments, the parameters of avalanches were collected over the entire stress range during the deformation and thus our histograms and exponents belong to stress integrated values [17,24]. In order to get the exponents from Equation (1), the PDF functions (calculated using logarithmic boxing) were fitted using a three parametrical nonlinear fitting by the Levenberg-Marquardt least squares method. In Figure 4b, the complementary cumulative distributions, CCDF, are also shown for comparison. It can be seen that the PDF is a nice straight line over three orders of magnitude of *E*. In addition, in Figure 4a,b the slopes of the two (PDF and CCDF) functions provide the same values *ε* = 1.45 ± 0.05 and 1.20 ± 0.15 (In Figure 4b the straight line just indicates the slope fitted according to the power law, and its value should be less by 1 than the slope of the PDF [39].) We also checked that the exponents estimated from the maximum likelihood fitting [40] agreed well, within the error bars, with those calculated from the PDF and/or CCDF (see also Figure 4c and the comments in Section 3.4 below). Similarly to (*P(E)*) from the size distribution functions (*P*(*S*)~*S*^−^*^τ^*) *τ* = 1.9 ± 0.1 and *τ* = 1.0 ± 0.3, values were obtained at low and high temperatures, respectively (see also Table 1).

**Figure 5:**
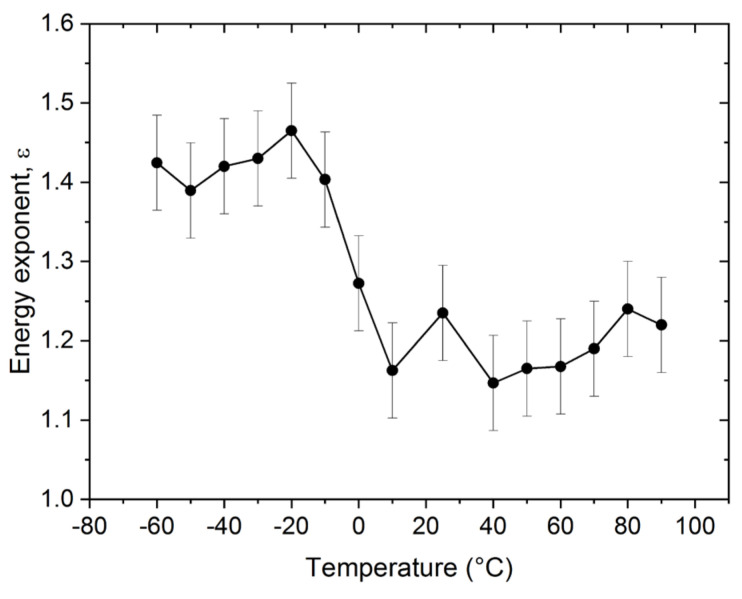
Temperature dependence of power exponent (*ε*) of the energy distributions.

Figure 5 shows the temperature dependence of the characteristic exponents of the energy distributions *ε*. It can be seen, that the energy exponent has a transition from *ε* = 1.45 ± 0.05 to *ε* = 1.20 ± 0.15 as the temperature increases from low values (below −20 °C) to high temperatures (above 50 °C).

Table 1 shows the average energy and area exponents belonging to the low and high temperature limits.

**Figure 6:**
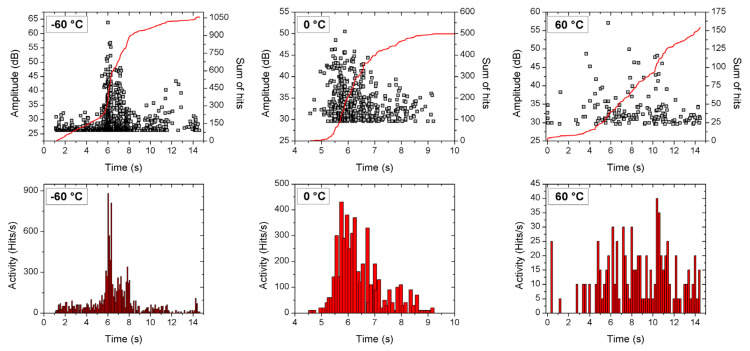
Acoustic activities as a function of time (the strain is proportional to the time) at different deformation temperatures.

Figure 2, Figure 3, Figure 4, Figure 5 and Figure 6 nicely illustrate that the characteristics of AE show a systematic dependence as the function of temperature in accordance with the transition from twinning to dislocation slip mechanism. Figure 4 demonstrates that the energy distributions indeed follow a power law behavior over about three orders of magnitude. Figure 3 especially illustrative showing that the total noise energy decreases by about two orders of magnitude during the transition between the two deformation mechanisms as well as the energy and area exponents decrease from 1.45 ± 0.05 to 1.20 ± 0.15 as well as from 1.9 ± 0.1 to 1.0 ± 0.3 (see also Table 1), respectively.

The results shown in Table 1 can be compared to the energy exponents determined earlier for plastic deformation of different metals. It is known in the literature [2,41] that, demonstrating the generic character of scaling laws (even if the twinning and slip avalanches could be discriminated from both AE weave-forms and populations [2]), the energy exponents is the same (*ε* = 1.6 ± 0.1) in hcp metals [41]. In addition, it was also shown that the testing temperature had an influence on the AE rate [13,41]; it was a function of the temperature, showing a maximum at 200 °C (see Figure 9 in Ref. [41]) in an AZ31 magnesium-based alloy (3 w% Al,1 W%Zn). This was interpreted in [1] as the result of the temperature dependence of critical resolved shear stress, which for $\left\{10\bar{1}2\right\}$ twinning and pyramidal slip decreased with increasing temperature [2]: the maximum at 200 °C could be a synergistic result of the two mechanisms. Furthermore, our results are also in line with observations that twinning produces AE signals of larger amplitude than dislocation slip [5] (see Figure 2).

Our results suggest some refinements to the above picture. The gradual changes with increasing temperature in the AE activity (see Figure 3 and Figure 6) show a gradual change between the two deformation mechanisms, similarly as it was observed in the AZ31 magnesium alloy [41] (the transition region is about 80 °C wide here). In addition, we got a definite change in the energy exponent. This contrasts the wisdom that the scaling law is so robust that the exponents are independent of the deformation mechanism. In order to strengthen this conclusion, we also investigated the following characteristics.

It can be shown that the relation between the avalanche size and its duration can be given as [42,43,44,45].

(2) $$S\sim D^{\gamma }$$

According to the definition of *S* and since the average amplitude $U_{av}=\frac{1}{D}\int_{0}^{D}\left|U\left(t\right)\right|dt,\;$ as well as using that the amplitude, *A*, (as a peak voltage amplitude) is a good measure of *U_av_* [24], one has:

(3) $$A\sim U_{av}\sim \frac{S}{D}\sim D^{\gamma -1}=S^{1-\frac{1}{\gamma }}$$

which gives the well-known relation *A~S^0.5^* [10,24] in MF approximation (*γ = 2*). Similarly, according to the definition of *E* ($E\sim \int_{0}^{D}U{\left(t\right)}^{2}dt):$

(4) $$E\sim U_{M}^{2}D\sim A^{2}D\sim A^{\frac{2\gamma -1}{\gamma -1}}\sim S^{2-\frac{1}{\gamma }},\;$$

where (3) was also used (for the derivation of relation $E\sim S^{2-\frac{1}{\gamma }}$ see also (Equation (7) of [43]). Thus, the values of *γ* can also be estimated from Equations (3) and (4).

Theoretical models (in the framework of a mean-field theory, MFT, or beyond [17,24,25,26,45,46,47]) predict values for the exponents *γ*, *τ* and *ε.* It was shown (e.g., in [26]) that *γ* (and thus *τ*) can vary between 1.56 and 2.0, respectively, for two different universality classes (for short- as well as long-range interactions). While for the stress integrated values of *ε* the MFT predicts *ε* = 1.33 as well as *ε* = 1.67 for not stress integrated and stress integrated values, respectively [17,24,47]. The exponents of probability density distributions belonging to stress integrated distributions are typically larger by about 25–33% than the corresponding not stress integrated exponents [24,47].

**Figure 7:**
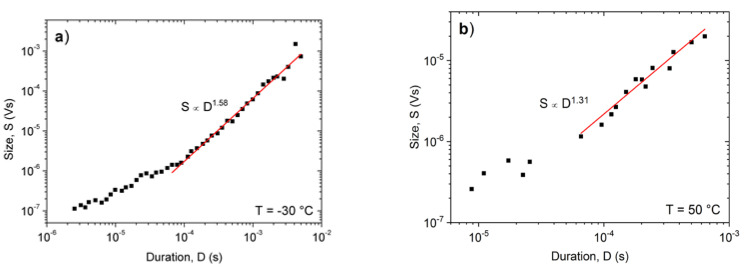
*S* versus *D* function at −30 °C (**a**) and 50 °C (**b**).

Figure 7 shows the *S* versus *D* plots at −30 °C and +50 °C. It can be seen that again the scatter is larger in Figure 7b, by a similar reason as it was in Figure 4a (much smaller numbers of hits at low temperatures: see also Figure 3). Nevertheless, it is clear that the exponents, *γ* are also different at high and low temperatures (*γ* = 1.56 ± 0.05 and 1.3 ± 0.1). Furthermore, the first parts, belonging to small values of *D* in Figure 7, are out of the region where the power fitting was made. The reason of this can be twofold: (i) these small values of *S* and *D* are sensitive to the choice of the threshold; and (ii) the effect of intrinsic absorption of the AE signal [29,30,31]. Indeed, it was shown in [29] that the signal intensity (defined as *I*(*t*) = *U^2^*(*t*)) is influenced by an exponential decay due to intrinsic absorption factor, exp(−*t/τ_a_*), where *τ_a_* is the characteristic time of absorption, and it was concluded that for short events (*D* < *τ_a_*) the AE duration is meaningless in terms of source mechanism [29]. On the other hand, it is expected that in the *D >> τ_a_* limit the above distortion effect of the transfer behavior of the system can be moderate or even negligible [30,48,49]. From our plot in Figure 7a, the time limit above which this effect can be neglected is about 100 μs (in [29] it was found that this value was also about 100 μs in a concrete material). In a very recent paper [31] the distortion effect of the acoustic emission device was nicely summarized. While the distortion effect considerably modified the duration time and the exponents of the scaling functions such as (2) at small parameter values (as it also can be seen in our Figure 7a), the exponents of the PDF functions of the energy and size were approximately invariant (i.e., their values were the same for the source signals and the measured voltage). This confirms that our results on the temperature dependence of exponents summarized in Table 1 reflect the changes due to the change of the deformation mechanisms.

On the other hand, the scaling laws (giving power law relations between *E* and *D, S* and *D* or *A* and *D* as Equations (2)–(4)) at small values of the duration times should reflect the behavior of the transfer function [29,30,31] and it can be a question how the exponents approach the exponents valid for the source even with increasing values of *D.* This will be analyzed after the treatment of the effect of driving rate.

### 3.2. Results at Lower Driving Rates

Since the characteristic exponents, and even the temperature at which the two deformation mechanisms changes, can be dependent on the deformation rate, we also carried out measurements at smaller driving rates (at $\dot{\varepsilon }=0.005,\;0.010,\;0.015$ and 0.025 s^−1^) at *T* = −10 °C, and at the original rate, at *T* = −30, 0, 25 (room temperature), 50 and 80 °C with more precise data collection. Each of the runs were repeated three times, and Table 2 contains the averaged values of the exponents *ε* and *γ* (obtained from the Equation (2)). The scatter between the exponents belonging to the repeated runs was about 15% for *ε* (i.e., about three times larger than the fitting error shown in Table 1). It can be seen from the data that there is no systematic dependence of *γ* on the driving rate.

Table 3 show the summary of result of the temperature dependence of the *γ* values, calculated also from plots *logE* versus *logD* or *logA* versus *logD* (see Equations (3) and (4)) as a function of the driving rate, obtained from fitting to long duration times (as shown in Figure 7).

It can be seen from Table 2 that no visible tendency on the rate dependence of γ. Interestingly, while the driving rate dependence of *γ* can be neglected, there is a small but definite driving rate dependence of the energy exponent, *ε*. This can reflect that the exponents of the probability density distributions can have a driving rate dependence [24], even for the same microscopic mechanism.

It is worth noting that we also checked that the *γ* values obtained at *T* = −10 °C for the above four, lower driving rates, which also agreed with the values belonging to the driving rate $\dot{\varepsilon }=0.15\;s^{-1}$ (i.e., the change of the transition themperature with the driving rates investigated should be small).

### 3.3. Effect of Distortion of the AE Signals Caused by “Ringing” of the Sample and AE Device during the Propagation of the Acoustic Waves

As we have seen the measured AE parameters can be polluted by the transfer effects, especially the duration time can be strongly distorted. Fortunately, our results on the energy and size exponents of the probability density functions (see Table 1) are invariant [31] and thus their change reflects the change in the mechanism. However, the fact that our *γ* values also show similar tendency as the energy or size exponents can be fortuitous. Thus, we re-consider the exponents describing power relations between different AE parameters (area, duration, amplitude, energy).

According to the results of [30,31,48,49] it is worth to make a clear distinction between the “true” values (not distorted by the convolution with the transfer function) of the area, duration time, amplitude and energy, let us denote by *S_AE_*, *D_AE_*, *A_AE_*, and *E_AE_* the corresponding parameters calculated from the AE measurements. Now it was shown in [30,48], that Equations (2)–(4) for the measured AE parameters are expected to be valid only above a certain value of the duration time (amplitude or area) (i.e., if the $\frac{C}{A_{Ar}}$ ratio is small enough (*C* is the threshold value) and if $\frac{\tau_{a}}{D_{AE}}\ll 1$). For the another limit ($\frac{\tau_{a}}{D_{AE}}\gg 1$), it was obtained that:

(5) $$D_{AE}=\tau_{a}\ln\left(\frac{AT}{C}\right)=\tau_{a}\ln\left(\frac{A_{AE}}{C}\right)\;\;\;\mathrm{if}\;D_{AE}\ll \tau_{a}$$

(see Equations (6) and (11) in [48]). Furthermore, can be shown, using again the arguments of [4], that:

(6) $$lnS_{AE}\sim \frac{D_{AE}}{\tau_{a}}+\ln\left[1-e^{-\frac{D_{AE}}{\tau_{a}}}\right]\;\;\mathrm{if}\;D_{AE}\ll \tau_{a}$$

**Figure 8:**
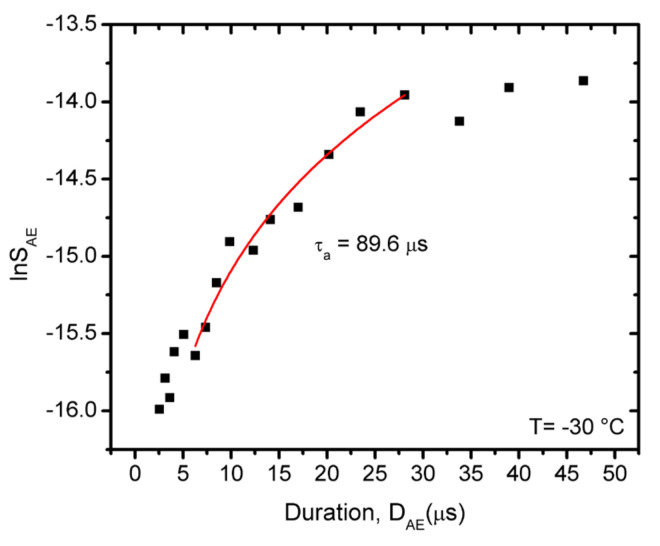
$\ln S_{AE}\;versus\;\frac{D_{AE}}{\tau_{a}}$ plot from points belonging to small values of *D_AE_,* which do not fit to the $S\sim D^{\gamma }$ relation in Figure 7a. From the fit (see Equation (5)) *τ_a_* = 90 μs was obtained.

**Figure 9:**
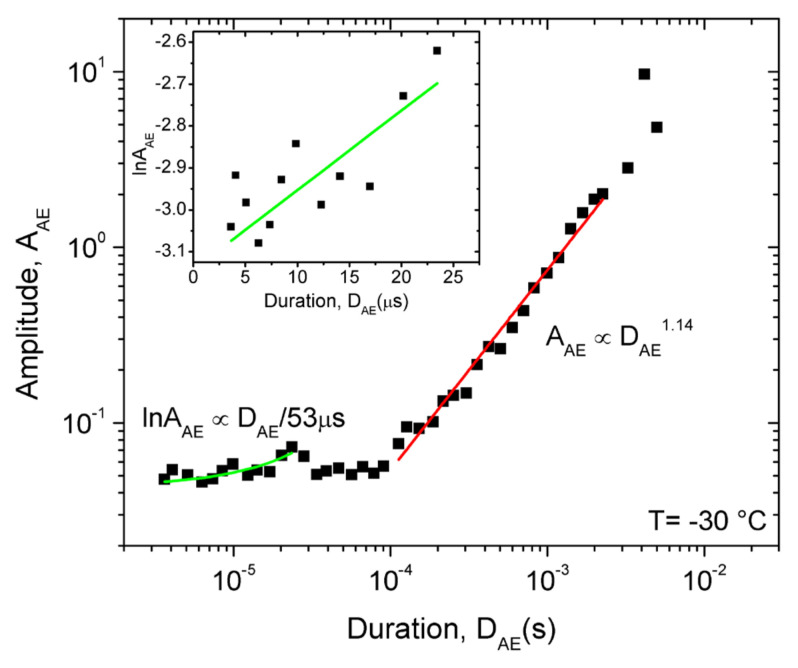
Relation between *A_AE_* and *D_AE_* at −30 °C. The straight red line fitted at large values of *D_AE_* has the slope (in accordance with Equation (3)) $\frac{1}{\gamma -1}=1.14,$ i.e., *γ* = 1.88 (see Table 3 as well). From the green curves fitted at short durations (see the insert as well, which show the ln*A_AE_* versus *D_AE_* function) *τ_a_* = 50 μs was obtained.

Figure 8 illustrates that the first part of the $\log S_{AE}\;\mathrm{versus}\;log\frac{D_{AE}}{\tau_{a}}$ function (see Figure 7a) indeed can be fitted by Equation (5) (in the following only low temperature measurements (−30 °C) will be analyzed due to the much better statistics of the data obtained), and the fitted curve provides *τ_a_* = 90 μs. We can similarly investigate, for example, the relations between *A_AE_* and *D_AE_* (see Figure 9).

### 3.4. Possible Fine Refinements of Data Analysis

Considering the shape of the ML curve in Figure 4c, it can be seen that it shows a definite maximum, which can be an illustration that our results contain superimposed power laws [50,51]. This can be the reason of the fact that the ML value of the exponent (Figure 4c) is a bit larger than the exponent obtained from the fit on Figure 4a. If we have two power laws with larger and smaller exponents, it might happen that, in the part after the kink, the curve cannot reach the value of the lower exponent [50]. This calls for further, more detailed analysis, using in parallel the so-called clustering analysis method [52,53,54,55] as well trying to distinguish between AE emission sources of different origin. This method uses a “spectrum-based” analysis in a frequency domain after a Fourier or wavelet transformation: different sources produce AE signals with different waveforms, and thus different power spectral density (PSD) functions. Therefore, AE signals originating from different sources will belong to different clusters, which are identified by the average PSD functions of the AE events. Since the values of *ε* for high temperatures are a bit smaller (even taking into account the scatter between different runs: Δ = ±0.15) as compared to the expected minimum value 1.33 predicted by the mean field theory, we have carried out a preliminary clustering analysis in the same way as it was described in [55]. It turned out that for high temperature runs, we could distinguish two different clusters with different energy exponents (of about 1.20 and 1.05). Since our acoustic activity was very low at high temperatures, more efforts are needed to clear up the reason of the obtained small value of *ε* (i.e., whether it is caused by the presence of different types of AE signals and/or if this is due to the combination of wild and mild fluctuations) [22]. Another option can be the application of the recently published method [56] to separate signals from different dynamical processes.

Another interesting question is related to Figure 8, showing the acoustic activity as function of time at 60 °C. Although the number of points is small, this figure suggests certain quasiperiodic amplitude peaks, which may be connected to an additional relaxation mechanism into plasticity [57]. In Ni micro-crystals, quasiperiodic bursts were observed [57] and explained by incorporating dislocation relaxation processes during the waiting time intervals between avalanches.

Investigations and detailed analysis of the above phenomena are out the scope of the present paper and would need a more sophisticated analysis of data and will be investigated in the future.

## 4. Conclusions

1. It has been illustrated that the characteristics of AE show a systematic dependence as the function of temperature when there is a transition from a twinning to a dislocation slip mechanism. The total noise energy decreases by about two orders of magnitude during the transition between the two deformation mechanisms, and the exponents of the probability density functions decrease with increasing time (Figure 7). For example, here the energy exponent decreased from 1.45 ± 0.05 to 1.00 ± 0.15 (see also Table 1), respectively.
2. The approximate values of the exponent of the scaling relation $S\sim D_{AE}{}^{\gamma }$ (obtained from the part belonging to large duration times, as suggested in [30,31]) have a similar temperature dependence as the exponents of the PDF functions.
3. It was determined that the *γ* parameter (as a parameter, characterizing the universality classes) was practically independent of the driving rate, illustrating that changing the driving rate, in the experimental range investigated here at a fixed temperature, did not caused a change in the deformation mechanism. The approximate values of *γ*, calculated from the exponents of the other scaling relations (see Equations (2)–(4)) are in good agreement with each other.
4. From the analysis of the distortion effect of the AE detection system, it was determined that the scaling Equations (2)–(4) indeed showed a curvature as predicted by earlier analysis of this effect [29,30,31,47,48]. This analysis was performed on measurements carried out at the low temperatures, where the AE activity was high enough to allow such an analysis. The parts belonging to large values of the duration times can be approximated by straight lines as predicted by Equations (2)–(4); for the average value of *γ* we obtained *γ* = 1.78. From parts at short duration times, the average attenuation time of the elastic waves was estimated as *τ_a_* ≅ 70 μs.

## Figures and Tables

**Table 1:** Average values of the characteristic energy and area exponents, *ε* and *γ*, according to Equation (1) at 0.15 s^−1^ driving rate.

	*ε*	*τ*
Twinning (low temperatures)	1.45 ± 0.05	1.9 ± 0.1
Dislocation slip (high temperatures)	1.20 ± 0.15	1.0 ± 0.3

**Table 2:** Calculated exponents as the function of the driving rate at *T* = −10 °C. At each driving rate, three measurements were carried out and the results are the average for the three runs. Results obtained at $\dot{\varepsilon }=0.15\;s^{-1}$ at −30 °C are also included for comparison.

Driving Rates at T = −10 °C (1/s)	*ε*	*γ* from *S* versus *D* (Equation (2))
0.005	1.10	1.77
0.010	1.14	1.63
0.015	1.20	2.05
0.025	1.30	1.79
0.15 (at −30 °C)	1.45	1.58
average	-	1.76

**Table 3:** Calculated values of *γ* at different temperatures.

T (°C)	S versus D	E versus D			A versus D		Average Values at High and Low T
		Average		Average		Average	
−30	1.58	1.70	1.84	1.86	1.88	1.77	1.78
−10 (at 0.025 1/s)	1.79		2.10		1.60		
0	1.58		1.60		1.96		
25	1.85		1.87		1.63		
50	1.36	1.39	1.26	1.30	– *	– *	1.35
80	1.42		1.34		– *		

* The scatter on these plots were too large to get any reliable estimate for *γ*.

## Data Availability

The data presented in this study are available upon request from the corresponding author.
